# Informational support and information-seeking during transition to parenthood: Baby Buddy Forward’s focus groups with pregnant women and new mothers in Cyprus

**DOI:** 10.18332/ejm/171360

**Published:** 2023-11-01

**Authors:** Ioanna Koliandri, Eleni Hadjigeorgiou, Maria Karanikola, Ourania Kolokotroni, Christiana Nicolaou, Veronika Christodoulides, Maria Papadopoulou, Christiana Kouta, Nicos Middleton

**Affiliations:** 1 Department of Nursing, Faculty of Health Sciences, Cyprus University of Technology, Limassol, Cyprus; 2 Birth Forward Non-Governmental Organization, Nicosia, Cyprus

**Keywords:** information seeking, pregnant women, mothers, decision making, informational needs

## Abstract

**INTRODUCTION:**

Relevant and accurate information during the transition to parenthood is vital for active participation in decision-making. The aim of the study was to gain an in-depth understanding of informational support and information-seeking practices among women in Cyprus during the transition to parenthood with a focus on the use of the internet and informed decision making.

**METHODS:**

Qualitative descriptive exploratory design of 12 focus groups with 64 participants representing different language-cultural groups served by the Baby Buddy Cyprus app. A topic guide covering expectations, experiences and practices guided the discussions. Data were analyzed using inductive content analysis.

**RESULTS:**

Seven themes and several subthemes emerged. In an ‘unsupportive system’, ‘void’ of informational support, pregnant women strive to have a ‘confident voice’. They find themselves ‘self-navigating in parallel worlds’ of formal and informal information, where the internet holds a prominent place. ‘Supplementing and filtering’, instinctively and selectively, results in a state of ‘doubt and faith’ towards the trustworthiness of the information but also healthcare providers. Effective communication with providers is needed to break the cycle, but seems dependent on the self-efficacy of the women themselves (‘art of communication’). Women ‘deconstruct and reimagine’ their experiences, often assigning responsibility on themselves for not having been better prepared.

**CONCLUSIONS:**

Women want control over decisions affecting their pregnancy. While the internet is a prevalent source of information, they value communication with healthcare providers and want direction. A shift is needed from current practices of unguided information-searching. Maternity healthcare professionals need to recognize this phenomenon, offer appropriate guidance, and support active participation in informed decision-making.

## INTRODUCTION

It is crucial for pregnant women and new mothers to receive accurate and timely information in order to have a healthy pregnancy and delivery^1^. In fact, the transition to parenthood has been described as a ‘window of opportunity’ for the establishment of health-promoting behaviors in general and the development of health literacy^2^. Health literacy refers to one’s ability to find information, understand, critically appraise and use that information effectively^3^.

Antenatal education (AE) should provide pregnant women with appropriate skills, knowledge and attitudes in various aspects of maternal–newborn health and care^4^. However, ‘traditional’ approaches to AE involve a series of lectures, seminars and/or workshops in the prenatal period^5^; yet, these commonly focus on pregnancy and labor rather than covering parenting skills in general, whereas attendance is not always high^6^. There are many ‘teachable moments’ beyond formal antenatal classes^7^, including personal contact during scheduled appointments with healthcare providers. However, these encounters may not be as rewarding, as they are often characterized by limited time^8^ while the experience is dependent on the quality of the user–provider communication^9^. Yet, the process of information exchange with healthcare providers is the key to actively engage in informed decision making^10^.

In order to meet their information needs, pregnant women may seek and collate information through various sources, such as providers, antenatal classes, books, family and friends, and nowadays almost certainly the Internet^11^. With no scarcity of information on the internet about pregnancy and childbirth, internet use is very prevalent amongst pregnant women^12^. However, there are issues with quality and, generally, women state that they have insufficient skills to evaluate information retrieved online^13^.

Currently, in Cyprus, antenatal classes are offered both in the public (open to all and free of charge) and private sector (commonly restricted to clients). In the public sector, midwives usually oversee the organization and coordination of the classes and various other healthcare professionals may be involved in delivering the content. However, there is no centralized regulation governing the structure and curriculum leading to variation in content but also availability of antenatal education options. It is important to note that the language of the antenatal classes in the public sector is primarily Greek, which poses a barrier for non-Greek speakers, while interpretation services are not provided. Beyond the public sector, some private clinics organize groups sessions for breastfeeding support and/or offer postnatal classes, with a fee to their clients, such as baby massage classes. A number of NGOs, such as the Cyprus Breastfeeding Association or Filotokos also offer breastfeeding support. The biggest change since the present study, as part of the establishment of the General Health System (GeSY), was that midwives can register as autonomous providers and women are allowed up to 6 visits to a midwife reimbursed by the system without a referral from a doctor. Nevertheless, and despite demand, a very low number of midwives have registered to date as autonomous providers with GeSY.

In this context and as part of the series of formative studies designed to develop the Baby Buddy Cyprus webapp, it was deemed important to understand the current information-seeking practices of women in Cyprus during the transition to parenthood. The Baby Buddy webapp is a free-access educational platform with daily text and videos to users from early pregnancy to the sixth month of the baby’s life (more information in the Supplementary file Material 1). The aim of this study was to gain an in-depth understanding of the informational support and information-seeking practices among women in Cyprus with a focus on the use of the internet and informed decision-making. Specifically, the research questions focused on women’s expectations (e.g. what are the learning needs), information-seeking practices (e.g. which are the main sources of information), the use of Internet in particular (e.g. how do they use internet as a source of information and how do they assess the validity of the information) and experiences in the context of informed shared decision-making. (e.g. what opportunities for informational support do they have; how do they describe the relationship and communication with healthcare providers; do they feel well informed to participate actively in decision-making).

## METHODS

### Study design and participants

Focus groups were employed in the current study to explore the information-seeking practices of pregnant women and new mothers. Focus groups were deemed preferable as group interactions have been found to encourage discussions among group members with potentially similar but also diverse perspectives, views and experiences; thus, providing a more comprehensive understanding of the phenomenon of interest^14^.

Twelve focus groups (n=64 women) with an average of 5–6 per group, pregnant (n=17) and new mothers (n=47), were conducted in five different languages with a diverse set of community groups served by the Baby Buddy Cyprus app: five in Greek, two in Turkish, Arabic, Russian, and one in English. The eligibility criteria were pregnant women (>2nd trimester) or new mothers (in the last 6 months, irrespective if first time parents or not), who gave birth or were planning to give birth in Cyprus. Participants spanned a wide range of nationalities (Arab, Australian, Austrian, British, French, Greek-Cypriot, Lithuanian, Russian, Syrian, Turkish, Turkish-Cypriot, Ukrainian). Table 1 provides an overview of the main sociodemographic characteristics of the focus group participants.

**Table 1:** Sociodemographic characteristics of the focus group participants (N=64)

*Characteristics*	*n*	*%*
**Age** (years)		
18–26	8	12.5
27–31	20	31.3
32–36	23	35.9
≥37	13	20.3
**Marital status**		
Married/cohabiting with father	58	90.6
Divorced	3	4.7
Other	3	4.7
**Education level**		
Primary	3	4.7
Secondary	7	9.9
Tertiary college	8	12.5
University	27	42.2
Postgraduate	19	29.7
**Nationality**		
Greek Cypriot	21	32.9
Turkish Cypriot	12	18.8
Other EU or British nationals	20	31.1
Syrian or other Arab speakers	11	17.2
**Number of births**		
0	17	26.6
1	29	45.3
2–3	15	23.4
≥4	3	4.7
**Type of last delivery**		
Vaginal birth	32	50.0
Cesarean section	12	18.8
Other	3	4.7
Pregnant/not decided	17	26.6
**Duration of last birth** (self-reported)		
1–4	19	29.7
5–8	12	18.8
≥9	16	25.0
Pregnant	17	26.6
**Place of last birth**		
Public hospital	20	31.3
Private clinic	24	37.5
Other	3	4.7
Pregnant	17	26.6

A convenience sampling technique was employed with an open call to register interest for participating. Printed and online information packs and other material were prepared in all target languages. Recruitment was achieved by disseminating on-the-spot information packs about the study at antenatal classes and maternity hospital/clinics as well as through Facebook posts and paid ads. These were disseminated both via the project’s own Facebook page as well as through collaborative efforts with other local partners and organizations related to maternal and child health (Table 2). Interest from women to participate was recorded through all recruitment pathways. With the exception of the two groups in Russian, which were mixed, separate focus groups were held with pregnant women and new mothers – an index is provided in Table 2.

**Table 2:** Information on communities of interest, recruitment methods, settings and procedures

*Focus groups (code/profile)*	*Recruitment methods*
Greek-Cypriot/Greek FG1/pregnant FG2–FG5/new mothers Turkish-Cypriot/Turkish FG1/new mothers FG2/pregnant Russian-speaking residents FG1–FG2/mixed Arab-speaking residents FG1/new mothers FG2/pregnant English speaking residents FG1/new mothers	● Information packs at antenatal classes ● Informational posters in maternity hospital, clinics and mum-baby centers ● Facebook posts and paid ads through the Baby Buddy Forward page ● Facebook posts shared through community organizations related to birth and maternal health (e.g. Birth Forward and Cyprus Breastfeeding Association) ● Facebook posts through community organizations working with specific community groups, with a focus on migrant women (e.g. Women Arab Club) ● Commercial partners in the private sector such as private antenatal or other mum and baby classes ● On the spot in seminars, workshops, conferences, fairs or other events related to birth and maternal health
***Focus group settings***	***Process and procedures***
Mum & Baby centers across different districts of the Island In areas controlled by the Republic of Cyprus: Nicosia Limassol Larnaca Paphos North of the Green Line with the Turkish-Cypriot community: Nicosia Kyrenia	● The invitation pack with an open call to register interest to participate was provided in all languages ● There was personal contact with all potential participants registering their interest to participate by phone ● The day and time of the meeting was arranged in advance. Different options of days (Wednesday/Saturday) and times (morning/afternoon) were provided to the potential participants and participation was confirmed by phone a few days before ● Participants were encouraged to bring babies or older children along and child-care was provided where necessary. Any last-minute drop-outs were almost all related to child care matters. ● Each session was facilitated by a native speaker and further attended by an experience facilitator and an observer for note-taking ● Sessions lasted 90–120 minutes. Refreshments and healthy snacks were provided during the sessions

There is no direct link between the bullet points listed on the right column and groups/settings on the left column. With regard to the recruitment methods in particular, all recruitment pathways stated above have been used for all communities of interest. The only notable exception was Arab-speaking women, whereby collaborative efforts were necessary through community organizations working specifically with Arab-speaking and migrant women.

### Data collection

Each focus group was facilitated by a native speaker of the language the focus group was conducted in. Prior to the study, all facilitators completed a structured training workshop covering skills, techniques, and good versus bad practices in focus group facilitation, along with mock practice sessions. All sessions were also overseen by an experienced facilitator and attended by an observer for note taking of non-verbal responses. Discussions were audio-taped and transcribed in the original language by each focus group facilitator, who subsequently translated the material into English for the purposes of the analysis. Due to the extensive material transcribed, no back translation was conducted by a second translator. However, the translators were familiar with the cultural nuances as well as the context of the discussion, which helped maintain the original meaning and intent of participants’ responses. Focus groups were held in Mum & Baby centers to provide a familiar, welcoming and child-friendly environment. Each focus group lasted about 90–120 minutes.

### Topic guide

Data were collected with the use of a semi-structured topic guide, shaped by the research team during a workshop following a structured iterative process. The process was guided by the conceptual model of health literacy^15^ and the logic model of developing parenthood preparation education programs^16^. The workshop participants were provided with a template sheet representing a cross-tabulation of the domains of the two models – an example and more information about the process is given in Supplementary file Material 2. Briefly, after an initial independent brainstorming session, the suggestions were gathered in a round-robin session, prioritized by consensus in terms of importance and relevance based on the aims, and finally organized in a logical flow with attention to phrasing to align with the purposes of the study. The final topic guide is presented in Supplementary file Material 3. This was first developed in English, and subsequently it was translated using a forward-backward translation method from English to the other target languages (Greek, Turkish, Russian and Arabic) to retain semantic equivalence.

### Data analysis

Translated material produced a total of about 155 and 174 A4 pages for the Greek and non-Greek language texts, respectively (with Times New Roman 12 fonts and 1.5 line spacing). Transcripts were analyzed together since the aim was not to draw inferences about differences between pregnant women and new mothers or between different community groups. Data were analyzed using an inductive content analysis approach^17^. In brief, the transcripts were read several times to ensure familiarity and coded independently in the first instance by three researchers (IK, EH, and NM). The initial codes identified were extensive; thus, there was an interactive process to compare coding and reach consensus regarding classification into categories. Through this process, a codebook with 37 categories (Table 3) was developed which was used to revisit the material to ensure that all meaningful information was classified, continuing iteratively until all text was captured and no other categories emerged. The classification of text was discussed and debated regularly to identify discrepancies and ensure process transparency. Decisions were made by consensus and, where necessary, any disagreement was resolved by involving a fourth researcher (MK). While moving from a manifest to a higher-order latent analysis, it became apparent that some identified themes/categories referred to and/or included elements related to the Health Literacy model, which partly underpinned the interview guide. While data were analyzed inductively, this presented an opportunity to connect some of the semantic patterns in the findings with the Health Literacy competencies conceptual model following an abductive analytical process^18^.

**Table 3:** Analytical presentation of themes and subthemes of the content analysis along with characteristic quotes

*Themes*	*Subthemes (category code book)*	*Quotes*
**(Un)Supportive system (‘The Void’)**	Healthcare system and structures	‘It’s probably not the doctors who are to blame but rather the system, because they are taught in such a way to be safe.’
	HPs’ skills, attitudes and practices	‘Some doctors are victims of their own success. They don’t devote enough time.’
	Unclear roles	‘What is their job? Does it fall under their practice? … it is the midwife’s job to inform on how to care for the baby, you know, lets separate [the roles] a little bit.’
	Lack of resources and services	‘Actually, they were not available in our area [referring to antenatal classes], I mean we didn’t have a chance to go to such things, I mean any things like this to make people aware, or something like that.’
	Gaps in care	‘To begin with, with the midwife, I never had an appointment, which I also consider to be a gap. And I consider that before the birth and after there should have been a continuation.’
	Own support system	‘So it was like I was a part of a community, but a virtual community anyway, so that was a lot of … I found a lot of support in that.’
**Self-navigating in parallel worlds**	Self-drive and direction	‘So I went out and researched where I could get that same experience.… so I went and did my own research.’
	Parallel worlds: Fragmented and conflicting	‘OK, I have trust in the pediatrician but at the same time, I am also updated by the groups [referring to social media mum groups], because what I feel is that there is a lack of correct information, we do not have overall information.’
	Multitude of sources	‘I was looking at things, and especially when I became a mother, from the internet and some books that I had seen, some seminars that I attended about breastfeeding.’
	Unwanted advice	‘They say it once, they say it twice, until you do it, and you go into a lot of.… that this thing everybody is talking about and this stresses me.’
**Supplementing and filtering**	Experience vs Εxpertise	‘With time though I understood that beyond the pediatricians and the internet, it’s also how your own child guides you.’
	The ‘specialist’	‘Each one is specialized in something, that is why during my pregnancy I was searching whatever was concerning me … I went to the specialist.’
	Filtering	‘I filtered through it very much because there was a lot of noise out there on the internet.’
	Selective (own choices)	‘You will take the information that suits you, then you see if it’s good for you or not. It means, what’s good for you might not be good for other women, because every woman has her own feelings and experience.’
	Perceived importance/ personal preferences	‘Sometimes I think, you know, when I look at reliable sources I want to see facts and it’s not that I find things that resonate in me that they are ok if they are opinion based as long as they are obviously the same as my opinions …’
**Doubt and faith**	(mis)Trust in HP	‘I didn’t trust the Internet with whatever has to do with my child. I consult only with my pediatrician.’
	(mis)Trust in information	‘So, I did not use the Internet anymore, because … there is a lot of rubbish there.’
	Worlds that don’t necessarily meet	‘I had my own issues, I was solid in the matters that … The gynecologist was at one end, I was at the other end, many times we did not agree, but because it is my baby, my body, I was very solid with my opinion.’
	Uncomfortable with uncertainty	‘I don't think it’s enough that I searched, even on my own, because I did a lot of things, read books, and went to suitable experts or anything … obviously it was not enough for me.’
	Disbelief and distrust	‘I want to say that you know absolutely nothing, and ignorance is like a sin in Buddhism. I agree with this, because then you are very easy to be manipulated.’
	Personal skills and circumstances	‘You simply need to know how to filter through, because there are things on the internet that are not correct.’
	Trust in self	‘You get lost, what to believe, who is right? This one or that? That's why at the end we believe ourselves, we do what convinces us, we trust our feeling.’
	Overwhelming process	‘Regarding this you are totally ignorant, to place the baby … and really you are in a constant confusion, I was in total confusion.’
**The art of communication**	Relationship with doctor	‘I had a very good midwife, we collaborated very well, but she was trained in that we change positions, because the particular midwife with the doctor with whom she was collaborating.’ ‘I actually had some issues with the doctor regarding our communication, we couldn’t communicate well.’
	To fight or not to fight?	‘Yes, really, you don’t feel like you have the power to say something, to express an opinion in general.’
	Ownership of care	‘But in the process of pregnancy I am responsible not only for myself but also for the child, so I am rather lack of this information and how it affects a baby.’
	Perceived role in participation	‘Usually, I asked their opinion on something… and they just gave me their opinion … but it wasn’t something for them to refute or adopt, it was just a reflection, a reflection of mine, given that I received that information.’
**Deconstructing and reimagining**	Internal reflections	‘I have a story that I want to share with you. I was pregnant with my third child, my daughter, so I had some kind of infection, and I drank some medicine that got mixed with my milk, and the doctor told me that I have to stop breastfeeding for 10 days. I felt very sad, and I brought formula and tried it for her, but she refused to take it. We handled it for 10 days, then I went to the doctor, he said to me you have no milk anymore, there is nothing. I was like what nothing? I went back home, I cried and cried and cried, unbelievable …’
	External comparisons	‘In Lithuania we have three years and we don’t have that problem/and for this reason … I am a lawyer and this is why I left work, to stay with the family, basically it is the mentality I grew up with …’
	Suboptimal practices	‘So, I went to the nursery to find one of the nurses to talk to the midwife or nurses on duty, and obviously to see my baby and I found they were giving her more formula, AGAINST my wishes. So, I was really upset with that.’
	Urgency vs Εmergency	‘It was where the people were kind of responding in a way where birth is a medical emergency, and I was, like, no it is birth, it is NOT a medical emergency. I feel healthy, I feel great, I feel good and I know this is the right way to do it.’ ‘At that moment if the doctor tells me “you know, now we have to do this” how can you question that? Even if you think about it.’
	Reimagining	‘I understand that I’m to blame for this, it’s not … I may not have searched for some things as much, to have an opinion. Because I considered, at the beginning I searched various things and then I said “if I search the internet or any other source, it means that I won’t have trust in the gynecologist” and so your experience with each specialist is more negative.’
**Confident voice**	Be prepared	‘So that every time I go, I have a plan that you know, we’ll do these tests in a month. In this period, you’ll feel like this, this will be happening, so I can be more prepared.’
	Do the ‘right’ thing	‘… and it’s really just to have somebody who says whatever you feel it’s right.’
	Options and choices	‘To be fair, for them it’s kind of automatic, they know what to do. But you, who goes for the first time, you have this experience for the first time, you need more information, I would have wanted him to tell me more, why do this, why … or to give me some choices, possibly.’
	Participate in decision making	‘We went out from a doctor’s office and I showed him the information from the Internet and he agreed with me. The information from the internet helped us to make a mutual decision.’
	Be in control	‘… I questioned it, and I stood up to her, and it’s almost, like she didn’t have a choice.’
	Safeguard rights	‘I understand his point, but I also have my rights.’

### Ethical considerations

Potential participants were informed about the study through an informational pack in all target languages. Both printed and online packs included a call to register interest in participating in a group discussion along with a list of specific days, times and locations. Upon registering their interest, potential participants were informed again over the phone about the objectives, requirements and process, when facilitators, who were native speakers, called to confirm participation. At the beginning of each session, there was a reminder about the purpose and procedures and all participants provided a signed informed consent in their native language, as per the standard format of the Cyprus National Bioethics Committee. Participants were ensured that they could terminate their participation at any given time without any explanation and were also provided with information about seeking support if participation triggered any unpleasant experiences. All participants provided consent in writing for audio-taping, transcribing and thematically analyzing the discussion. The study protocol and related material was duly approved by the Cyprus National Bioethics Committee (EEBK EΠ 2018.01.124).

## RESULTS

**Figure 1:**
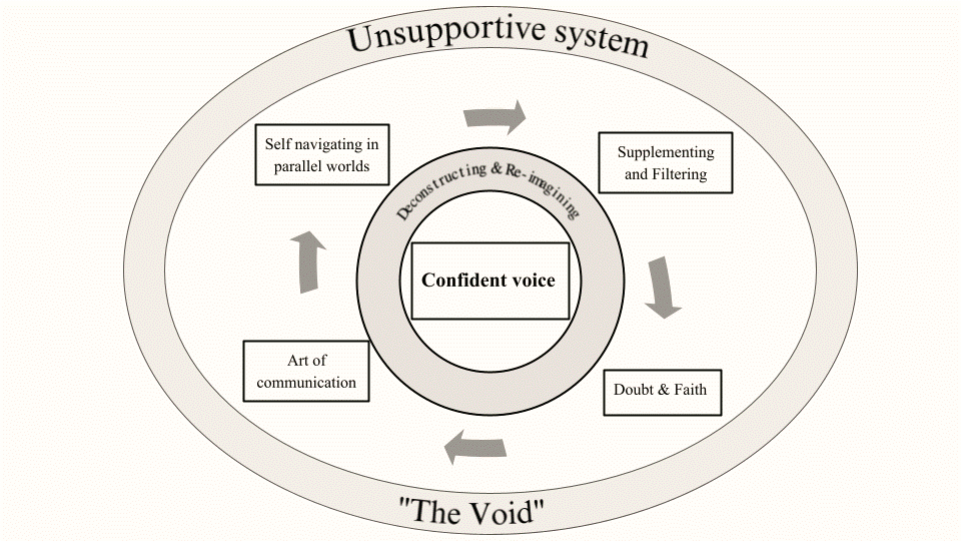
Schematic presentation of the main themes of the inductive content analysis

Seven themes (Figure 1) and several subthemes emerged (Table 3): 1) Unsupportive system (‘The Void’), 2) Confident voice, 3) Self-navigating in parallel worlds, 4) Supplementing and filtering, 5) Doubt and faith, 6) Art of communication; and 7) Deconstructing and reimagining.

As mentioned earlier, the conceptual model of the Health Literacy competencies (Access, Understand, Appraise, and Apply) was used to connect four of the seven identified themes which represent the processes of informational support. Thus, introducing elements of abductive analysis, ‘Self-navigating in parallel worlds’ represents the concept of Access to information, while Understanding is loosely represented by ‘Supplementing and filtering’, Appraisal by ‘Doubt and faith’, and Apply by ‘Art of communication’.

An integrated narrative structured along the main themes follows, illustrated with quotes.

### Unsupportive system – ‘The Void’

Women spoke about an unsupportive system when it comes to informational support. It was often related to the lack of time during appointments:

*‘[…] you wish they could just give you more time, to let you take a breath, just some time!’* (Arabic FG1)

Lack of time was not the only issue; a number of participants suggested that even when there was some information exchange with healthcare providers, this was often incomplete:

*‘[…] the physician must provide this information, but it doesn't happen, the information is incomplete’* (Greek FG2)

and one-sided, not providing them the chance to actively participate in the discussion:

*‘[…] And they don’t give you a chance to talk…’* (Arabic FG2)

Descriptions sometimes reflected health professionals’ attitudes. Characteristically, one woman described this scene where the only reason she understood some things was because she was listening when a midwife was discussing her with the student midwives without including her in the conversation:

*‘ … one of the students palpated my abdomen and discussed about me with the midwife …and I listened, this helped me understand many things …’* (Greek FG1)

Descriptions also reflected lack of information on available services:

*‘If I knew there were classes I would have attended.’* (Russian FG2)

*‘[…] I don’t know, is there somewhere where you have appointments with midwives?’* (Greek FG3)

Even women who attended antenatal classes commented on how they were not informed about them early enough, that these classes were offered infrequently and were not always useful:

*‘I had been to Makario [referring to antenatal classes], but I also learned about it in the middle of the period, because they start every six months. Some were very interesting, some less …’* (Greek FG2)

The lack of an established Community Midwifery system left women feeling alone and vulnerable, not knowing what to expect:

*‘There is really nobody checking on the mother and how the mother is going and what the mother can expect after birth.’* (English FG2)

Women felt even more unsupported in the postnatal period:

*‘… after pregnancy, there's a big void.’* (Greek FG4)

The term ‘void’ was in other participants’ descriptions. One participant described the experience as finding yourself in a ‘void’ where any information, no matter the source, is a bonus:

*‘… considering you are in the void, whatever you learn is a bonus, always a positive.’* (Greek FG4)

### Confident voice

In this unsupportive system, women were struggling to have a ‘voice’, to raise questions, to communicate concerns and to express opinions. They understood that being well-informed was necessary for that voice to be confident but wondered how someone achieves that:

*‘How does someone get that voice? A confident voice? … especially if you are a first-time mum’* (English FG1)

The need to have a ‘confident voice’ seemed to take different nuances for participants. For some, it was presented in descriptive terms of just ‘needing to know’ what to expect and to be prepared, while for others it served the need to be appropriately supported in order to negotiate the complexity of information, to do the ‘right’ thing and make the ‘right decisions’:

*‘A mother today needs to have thirteen degrees … to be able to take the right decisions.’* (Greek FG4)

For others, the descriptions were driven by the need to participate actively in decision-making, to understand why things are done, whether there are alternatives, knowing their options and having choices:

*‘You need more information … why do this, why…or to give me some choices.’* (Greek FG5)

The value of information, described as the ‘truth’ or the ‘facts’, was at the core of both the need and the process of effectively participating in shared decision-making:

*‘You just need to have access to all the information … so that you can make your own decisions … by knowing what choices are available … by presenting all the information, the truth, the facts.’* (English FG1)

Recognizing that currently this is not the norm, participants felt that this process is dependent on the women themselves rather than initiated by the healthcare providers:

*‘Women really WANT to have choices, to seek out choices about medical issues. You can't expect to be presented with choices, which should be the norm, which should be the way it's done.’* (English FG1)

Nevertheless, the need to actively participate in decision-making was not evident in everyone to the same degree, and some women seemed to place their trust in the decisions the physician made for their care:

*‘I am generally satisfied with … the decisions the doctor takes.’* (Greek FG5)

In contrast, some participants perceived the lack of information as threatening:

*‘They (women) need to protect themselves …’* (English FG1)

and connected the need to be well-informed with safeguarding their rights:

*‘I believe that a woman should have the right to choose according to how she feels comfortable.’* (Turkish FG1)

### Self-navigating in parallel worlds

Women referred to information coming from multiple sources, from providers, friends, family, other mothers, and almost always from the internet, which seemed to be at the core of information-seeking. Women reported navigating on different websites or watching videos on YouTube, often describing the need to compare across different sources ‘to be sure’:

*‘Personally, I go on the internet, I look for information on the internet or from mother groups, …, from books and from other mothers.’* (Greek FG2)

*‘We like diversity, so as I said, we look at more than one website. You see information or you read something, you want to be sure, because there is a lot [of information] and lots of videos.’* (Arabic FG2)

But often, information is ‘unwanted’ as it comes in the form of opinions, suggestions and advice from everybody around them:

*‘Everybody, everybody knows … we deal with this all the time.’* (Greek FG1)

In these parallel worlds of information, the process of retrieving and assessing information was described as selfdirected without any guidance:

*‘If you have a drive, you will find something, but for something like that, where do you go to ask?’* (Greek FG2)

The need was perceived as dependent on the woman’s personality and own self-driven interest:

*‘I would like to say, the mother is obliged to research, and it may also be part of each mother’s character, how far she wants to research, research everything.’* (Greek FG1)

Often, internet and health professionals are two parallel worlds that do nοt meet. For example, instead of discussing with their provider, women try to verify information the provider gave them on the internet:

*‘To find reliable data, you are checking, and you do a match, you match the information with the information your doctor gave you …’* (Greek FG1)

Women seem to place the responsibility of being ‘informed’ on themselves while guidance from providers does not feature in their descriptions, even when it comes to antenatal classes. Characteristically, one participant who attended classes used the term ‘discovered’ (i.e. a self-driven process rather than a recommendation by a professional) and considered herself lucky for finding out about this opportunity early enough to be able to attend the full series.

### Supplementing and filtering

With lack of guidance, women are self-navigating in a multitude of sources, being unsure what is valid and what is not. Some women choose to pick and supplement from bits and pieces and others cross-check information across different sources to reach conclusions. This process of ‘Supplementing and filtering’ takes certain skills that not all women have:

*‘To begin with you need to have a lot of time and you need to know how to filter, which is not always that easy, considering you don’t have someone.’* (Greek FG4)

While cross-referencing the information seems to be a popular ‘technique’, in many cases, women simply described the process of filtering information as intuitive:

*‘I always filter what I see, and I reach the final conclusion myself…’* (Greek FG5)

Women perceived this abundance of sources as chaotic and the process stressful. While recognizing problems with the quality of the information online, they often felt it was the only choice:

*‘I was worried to death actually, the only place I could get information about this was the internet and as they say [agreeing with the other participants in the focus group], there is a lot of misleading information.’* (Turkish FG1)

Interestingly, some women felt that they needed to filter even the information they receive from their healthcare providers by using the internet to verify what the doctor told them:

*‘We have reached a point where we consider filtering what scientists tell us. Because they are not always thinking scientifically…’* (Greek FG5)

This process extends to seeking advice from informal sources (e.g. Mum groups and forums) and cross-referencing what the ‘experts’ say with the personal ‘experiences’ of other mothers, which many women value (‘Expertise vs Εxperience’):

*‘It's not that I don't trust the pediatrician, but I try for some things to be informed from elsewhere, and from other experiences of other mothers, and from groups, … on Facebook, … it is very helpful, because … you see different approaches.’* (Greek FG1)

*‘I mean when we ask the experts [referring to doctors], we find out how misleading the information is. I think the best thing to do is to talk to people with experience [referring to other women who gave birth].’* (Turkish FG1)

Yet, others who are overwhelmed with information coming from ‘parallel worlds’, choose to seek information only from health professionals. However, in their descriptions they do not seem to always place their trust on a single health professional but, depending on the matter or concern at hand, they prefer to seek out advice from ‘the specialist’:

*‘I prefer to go to a specialist as well. Even if one doctor tells me, I don't know, ‘do it like this’, okay… maybe the nutritionist is more specialized or the endocrinologist is more specialized, I'll prefer to go to the more specialized one.’* (Greek FG5)

‘Perceived importance’ of a topic seems to be a driving force behind the act of seeking information. However, the process of filtering information seems ‘selective’. Descriptions highlight an element of choosing information (or ‘adopting’ as per the actual term used by one of the participants):

*‘I was just adopting … if something was close to my opinions on nutrition, I adopted it.’* (Greek FG3)

The process of ‘adopting’ information is sometimes driven by own personal views and opinions; sometimes taking the shape of seeking information in order to confirm their viewpoint:

*‘Eventually, you will take the information that suits you …’* (Arabic FG1)

*‘I want to read facts that back up the opinions that I like.’* (English FG1)

### Doubt and faith

This process of seeking (accessing) and making sense (understanding) of information often leaves women in a state of ‘Doubt and faith’; both with reference to the information itself but also in terms of the healthcare providers. For many, the overwhelming amount and doubtful quality of the information meant they preferred to place their ‘faith’ on the doctor:

*‘I was seeking the advice of my doctor only, the doctor exclusively.’* (Greek FG4)

This was not the case for all participants. Some were more skeptical and felt they could not always trust their provider. One participant identified inconsistencies in the information between different providers:

*‘That is to say, even the information you receive from health professionals on various subjects, in essence, don’t agree.’* (Greek FG1)

The realization that the information on the internet was not always reliable was prevalent:

*‘I do not think there’s much correct information on the internet, there are many misleading stuff.’* (Turkish FG2)

while the process was described as stressful or even harmful:

*‘There was a lot of information that ultimately ended up being harmful to me.’* (Greek FG5)

This process often led to an overwhelming state of ‘doubt’, leaving women feeling confused:

*‘Really you are in a constant confusion.’* (Greek FG1)

and anxious:

*‘So, I was getting stressed, meaning it created a vicious circle for me, and I was really suffering.’* (Greek FG1)

This ‘confusion’ is not only the result of the challenging task of filtering information but also the reluctance of healthcare providers to engage effectively in discussion, a process that sometimes was stressful in itself:

*‘The gynecologist was at one end, I was at the other end, many times we did not agree.’* (Greek FG1)

At the same time, it emerged that women were perhaps uncomfortable with uncertainty, continuously searching for the ‘right’ information, often resorting in trusting their own intuition:

*‘You get lost, what to believe, who is right? This one or that? That's why at the end we believe ourselves, we do what convinces us, we trust our feeling.’* (Arabic FG2)

### Art of communication

In this repetitive (‘vicious’) cycle, the personal relationship with the healthcare provider was crucial but the ‘art of communication’ takes effort, and from many of the descriptions it appeared that often it was the women themselves that had to make the biggest effort. One participant, describing the need to make appointments worthwhile, talked about the need to be adequately prepared. In order to support her choices, she describes how she had to do extensive research before appointments in an effort to stand on equal ground with her healthcare provider:

*‘… to be able to say what you want and justify it, justify it with a little bit of evidence you can present to a scientist …’* (Greek FG3)

Descriptions suggested a wide variation in the quality of relationship with providers. Some of the women described having good communication with their provider, even though many reported that they had visited several doctors before choosing:

*‘I tried with more than one doctor… some of them were too much in a hurry, whether pediatricians or obstetricians, it's the same thing.’* (Arabic FG2)

Easy access to the doctor and their responsiveness to requests seemed to be for many of the participants a determining factor of their satisfaction with the doctor:

*‘We found a common language very quickly, we had a good connection.’* (Russian FG1)

*‘… I prefer to call my doctor … he often resents me because I bother him, but he answers.’* (Greek FG3)

Not all women have that relationship with their providers:

*‘I keep a distance … I won't call and ask for information. I'm not comfortable.’* (Greek FG5)

and many women were not as successful in making appointments worthwhile or getting the answers they need, even if they prepared in advance:

*‘If you prepare somehow, with a list of questions … they may answer them.’* (Greek FG1)

For non-Greek speaking women, getting much out of the appointment was even harder:

*‘… maybe it’s the language … or maybe it was their character, they were rushing, I was like: OK, I NEED TO DISCUSS [with emphasis].’* (Arabic FG2)

In cases of problematic communication, women were faced with the choice ‘To fight or not to fight?’. Often, terms such as ‘challenging’ and ‘negotiating’ were used in the descriptions, as if referring to a debate or an argument. Some even described the process as weary, often choosing to give up the ‘fight’. Terms such as ‘giving up’ or ‘bowing’ to the healthcare providers’ decision appear in the descriptions:

*‘So, at some point I gave up challenging, as I always saw, like you [addressing another participant], I was the only one trying to question procedures, and so, I just gave up.’* (English FG1)

*‘The decision of giving formula to my daughter … it was her decision [referring to the pediatrician], and I regretted that I bowed to her decision.’* (Arabic FG2)

Some women seem to try to negotiate in order to safeguard their choices. Descriptions again suggest that this is a ‘struggle’, rather than an alliance, in order to experience pregnancy or childbirth on their own terms:

*‘You just have to be really strong to … to … to … to hold your ground.’* (English FG1)

Some of the women’s descriptions suggest that they perceive the process of communicating and safeguarding their choices as a combination of strength of conviction and character on one hand, but also how well-informed they are; a process, nevertheless, that they expect to undertake on their own:

*‘That stuff is really really difficult to negotiate with them unless … you really really need to know your stuff.’* (English FG1)

### Deconstructing and reimagining

While recounting their stories, women seem to be deconstructing the experience to its elements (e.g. ineffective communication) and often referring to specific events which point to ‘suboptimal practices’ (e.g. non-medically justified cesareans, episiotomies or unnecessary interventions and procedures, etc.). At the same time, they were reimagining how else it might have been (‘in retrospect’, ‘in hindsight’), often reproaching themselves for not being adequately prepared to handle situations differently.

For some women, this created feelings of regret, leaving them feeling that they share part of the blame because if they had the right information, if they ‘had done more research’ and ‘looked into it a bit more’, their experience may have been different:

*‘Maybe I could've … I don't know, done some research and see how I could have gotten past that.’* (Greek FG5)

Other than internal reflections, there were external comparisons between the Cypriot healthcare system and abroad, from experience or things they have heard:

*‘While abroad, midwives do play a significant role from what I know. In some countries anyway.’* (Greek FG5)

*‘But in Syria I feel that it’s different, for example in my mum's case, my aunt's, I feel it's different, they give you all the information, whether in a hospital or a clinic.’* (Arabic FG1)

In the over-medicalized setting of Cyprus, being well-informed and empowered to counteract pressures was described by a number of participants as crucial:

*‘For women who feel … who are not, who didn't have the access to the information that I had, they hear this terminology HIGH RISK and emergency, and all of this, so they honestly feel like you’re doing the best thing for your baby by choosing to have medical intervention, is a privilege and it should be a choice, you know.’* (English FG1)

## DISCUSSION

Overall, the findings suggest that women want control over decisions (‘confident voice’) affecting their pregnancy and childbirth. However, current arrangements do not support informed decision-making (‘the void’) and the quality of communication with healthcare providers (‘the art of communication’) is not always unproblematic. Currently, the process of information-seeking seems self-directed (‘self-navigating in parallel world’) and, with lack of guidance, women ‘supplement and filter’ bits and pieces from several fragmented sources, both formal and informal and almost always the internet. The result is a state of ‘Doubt and Faith’ whereby effective communication with healthcare providers is important but often seems dependent on the self-efficacy of the women themselves.

Primarily, the study findings suggest that formal antenatal education does not address the women’s needs; a finding that has been previously described in the international literature^19,20^. The effectiveness of ‘traditional’ arrangements has been questioned both in terms of the extent to which they actually address the real needs and wishes of expectant parents^4^ as well as in terms of their inclusiveness with evidence of social inequalities in access, learning opportunities, and health outcomes^5,21^. In the case of this study, it seems that there is also a problem of access, as many women did not even know such opportunities were available. Previous studies have suggested that only one in three women in Cyprus attend antenatal classes^22,23^.

Pregnant women often turn to the internet for informational support^24^, other than easy access, it may also be a source for emotional support through forums and chat rooms^25^. Studies have suggested that women tend to cross-reference sources to verify information regarding their accuracy^26^; however, it is indicated that women lack guidance and direction from their providers on trustworthy sources on the internet^27^. Interestingly, instead of discussing information retrieved from the internet with their provider, women tend to double-check the information given by their provider on the internet^12^. This phenomenon was also confirmed in the present study. Lack of time is often cited^28^, but it also seems that reluctance of healthcare providers to engage and discuss such information, instead choosing to discourage the use of the internet, is a barrier^29^.

Findings from our study also highlight the significant challenges in the communication and relationship between women and health professionals; this is consistent with other studies where the communication with health professionals often do not meet the expectations of women^30^. This calls attention to the need for continuous professional development to strengthen communication skills and competencies of healthcare providers, particularly their willingness and ability to effectively engage and actively encourage participation of women in decision-making, with personalized attention to circumstances and needs^31^. In the case of Cypriot midwives in particular, a parallel Baby Buddy-related study provided evidence to suggest that, while they identify antenatal education as a core function of their professional role, beyond the formal setting of the antenatal class, there are several barriers in effectively assuming an educational role as part of routine clinical practice^32^.

Pregnant women and new mothers in this study expressed their need to have a ‘confident voice’ through their journey to parenthood. The quality of the relationship with the health professional is an essential element of the shared decision-making process which consequently empowers women^33^. Information empowers women to make informed decisions, counteract uncertainty, address fears and strengthen trust in their knowledge about their pregnancy and birth experiences^34^. Insufficient information and information exchange between pregnant women and healthcare providers do not promote shared-decision making which is essential in promoting autonomy and informed decisions, while discussing options with their health provider makes them feel valued and empowered^35^.

### Strengths and limitations

The number of focus groups was pre-determined due to resources. While data saturation was reached, it is uncertain whether theoretical saturation was achieved, especially for the non-Greek-speaking groups since a smaller number of sessions were conducted in the other languages. However, the aim was to provide a unified understanding of women’s experiences and it was beyond the scope of this analysis to draw comparisons or differences between the various groups. In light of the current description of the phenomenon, further research focusing exclusively on specific community/language groups may uncover whether certain identified concepts are more dominant in some subgroups or even identify new concepts.

Furthermore, due to convenience sampling, it is likely that participants share common experiences. The fact that only one in four of the women in the sample had a cesarean section (while the national rate of C/S is over 60%) is indicative of the lack of representativeness due to the voluntary nature of participation. A more heterogeneous group may have identified different aspects of the experience; thus, limiting the generalizability of the findings. However, the large sample and purposive sampling in terms of different community groups, public–private sector and socioeconomic status enhances the sample heterogeneity. In addition, the study focused on the experiences of pregnant women and new mothers; thus, not capturing the perspective of partners/fathers. It would be essential for future research to incorporate their experiences and role with regard to information-seeking and decision-making processes during the transition to parenthood.

Due to the large number of participants, no feedback was obtained from the participants for the purposes of confirmation and recognizability of the experience. Nevertheless, the resonance of the phenomenon, reasonableness of its presentation and readability of the narrative were confirmed by presenting the findings at the stage of analysis to the Birth Forward Board of Directors, a local NGO advocating for improvement in birth practices in Cyprus, consisting of health professionals and parents alike. Furthermore, in accordance to the Participatory Research and Learning principles underpinning the wider project, the findings were presented during a plenary session to delegates at the launch event of the Baby Buddy Cyprus webapp, as a means to promote reflection among maternal–child healthcare providers, the majority of whom were midwives. This contributed in cultivating a raised group consciousness with regard to the educational role of healthcare providers in light of women’s experiences, which was evident at the group discussion sessions that followed.

Finally, the transferability of the study findings is supported by the exploratory mixed-method design of the study itself, since the qualitative study subsequently shaped the design of a quantitative survey (not reported here) to explore further important concepts that emerged from the qualitative results, such as the quality of communication with providers and the degree of participation in decision-making.

## CONCLUSIONS

Women stated their need for informational support and effective communication with their healthcare providers. However, the current arrangements do not seem to be supportive of women’s participation in informed decision-making, which often results in lack of trust not only towards their providers, but in the healthcare system in general. Pregnant women in Cyprus want to have control over decisions affecting their pregnancy (‘confident voice’). While they turn to the internet for information, they value communication with their providers and want direction. Maternity-child healthcare providers need to recognize the phenomenon rather than ignore it. They need to recognize their educational role, engage and communicate effectively during routine appointments, be aware of what information is out there, shortlist good quality digital resources to suggest to their clients, offer appropriate guidance, and support shared decision-making.

## Data Availability

The data supporting this research are available from the author on reasonable request.
