# Supplementary material for: Informational support and information-seeking during transition to parenthood: Baby Buddy Forward’s focus groups with pregnant women and new mothers in Cyprus
Source: Eur J Midwifery. 2023 Nov 1;7:29. doi: 10.18332/ejm/171360 (PMC10619000; doi:10.18332/ejm/171360)
Supplement: Supplementary file 1 [file EJM-7-29-s1.pdf]

Supplementary Material 1: Information about the Baby Buddy Forward project

The present study is part of the wider "Baby Buddy Forward" project (full title: "*Building health literacy around pregnancy, birth and early life of the infant by developing a unified, research-informed and socially-inclusive prenatal and postnatal web-based education programme for parents-to-be and new parents*"), co-funded by Erasmus+ (2017-2020), under the scheme KA2 - Cooperation for Innovation and the Exchange of Good Practices. KA204 - Strategic Partnerships for adult education.

Following Participatory Action Research principles, the Baby Buddy Forward project assessed the cross-cultural transferability of the Baby Buddy app, developed by UK by project partner Best Beginnings to the highly medicalized and decentralized Cypriot setting. Conceptually, the wider project is underpinned by the *Respectful Maternity Care* (RMC) Framework (WHO, 2016). Other the need for good quality perinatal care with effective clinical practices, the RMC framework states the importance of relevant and timely information and psychosocial and emotional support, by knowledgeable, supportive and respectful health-care practitioners with good clinical and interpersonal skills, within a well-functioning health system, to support decision-making.

The main deliverable of the Baby Buddy Forward project was the Baby Buddy webapp (https://www.babybuddy.com.cy/) – a free platform delivering text and video messages to registered users every day from early pregnancy up to the sixth month of the baby's life.

As part of the series of sequential formative qualitative and quantitative studies designed to deliver Baby Buddy Cyprus, it was deemed important to gain an in-depth understanding of the current information-seeking practices of pregnant women in Cyprus, with a focus on the use of the internet as an information source, as well as the quality of communication and informational support from healthcare providers.

The series of focus groups reported here was the first stage of an exploratory mixed-methods study. The qualitative study provided an in-depth understanding of the information-seeking practices and related determinants, in order to guide the design of the subsequent quantitative survey to further explore the main concepts that emerged from the qualitative study, such as perceptions about the use of internet for information in pregnancy and the quality of communication with healthcare providers.

Supplementary Material 2: Mapping of questions to conceptual and logic model in relation to “teachable moments”

The topic guide for the focus groups was shaped by the research team during a training workshop following a structured iterative process. The process was guided by the conceptual model of health literacy as described in four sequential competencies of access, understanding, critical appraisal, and use/application of information by Sorensen et al (2012) as well as the logic model of developing parenthood preparation education programme (Gilmer et al, 2016) with reference to potential sources of information and “teachable moments”; for instance, personal appointments with healthcare provider, antenatal classes, internet, other sources and resources of information, including the person’s social circle.

Briefly, the process entailed the following steps:

- Workshop participants were provided with a template sheet representing a cross-tabulation of the domains – see table below
- After an initial independent brainstorming session, all suggestions were gathered in a round-robin session
- Subsequently, they were prioritized by consensus in terms of importance and relevance based on the aims
- Selected questions were organised in terms of core (essential) questions vs prompts and probes,
- Lastly, the questions were reviewed with attention to phrasing and organized in a logical flow making sure they align with the purposes of the study
- The final topic guide was subsequently translated using a forward-backward translation method from English to the other target languages (Greek, Turkish, Russian and Arabic) to retain semantic equivalence.

**Health Literacy (conceptual model) Sorensen et al (2012)**	**Parent education implementation chain (logic model) Gilmer et al (2016)**	**User-Provider Interactions (encounters)**	**Antenatal/ parenthood preparation classes**	**Internet or traditional sources and resources (e.g. leaflets, books, etc)**	**Friends, family, and wider social network**
		**"Teachable moments"**			
**<u>Access:</u>**<br>Learning needs & Opportunities	**A.** Perception that there are knowledge gaps; hence, "learning needs":	• Perceptions of learning needs?<br>• What topics & issues matter to you?<br>• What opportunities are there for informational support?			
**<u>Understand:</u>**<br>Sources<br>Content<br>Barriers.<br>Skills	**B.** Design programmes to meet perceived "needs" (felt and expressed)	Opportunity to express needs?<br>- Time?<br>- Interaction?<br>- Sharing?	Do you attend? If not, why?	Other sources?<br>- Which?<br>- How often?<br>- What do you search for?	Who do you talk to?<br>- Partners?<br>- Parents?<br>- Other mums?
**<u>Critically appraise:</u>**<br>Relevance & Trustworthiness	**C.** Delivery of the programme: fidelity, attendance, satisfaction	Experiences from user-provider interaction?<br>- (Un)Satisfied needs?<br>- Perception of internet use/ reactions of HP?	- What's not discussed?<br>- What is lacking?<br>- What could be done differently?	- Trustworthiness of information?<br>- How do you assess?	- Why do you turn to them?
**<u>Use/Apply:</u>**<br>Usefulness & Outcomes	**D.** Efficacy: value, confidence, new skills<br>**E.** Effectiveness: choices and behaviours<br>**F.** Positive outcomes of parenting behaviours	• What is most useful/ what is not? Why?<br>• How to apply this knowledge? What skills does it provide?<br>• How can user-provider communication be supported?<br>• What is most helpful in terms of decision-making?			

Supplementary Material 3. Topic guide for the focus groups

Topic (as related to Aims/ Objectives)	List of potential questions: Essential, Prompts (promote discussion) & Probes (in-depth)	
**Experience (focus on informational support)**	**(1) Now that you are pregnant [When you were pregnant], when you want [wanted] more information about something, what do [did] you do?**<br>● How did you prepare yourself? Who or where do/did you turn to for information?	**Essential**<br>**Prompts**
**Opportunities (in addressing learning needs – "teachable moments")**	**(2) About antenatal/parenthood preparation classes, what do you think of those?**<br>● Did you attend any classes or seminars during or after your pregnancy? Why? Why not?	**Essential**<br>**Prompts**
	**(3) What about at the appointments with your provider (gynecologist, midwife, pediatrician, other) – can you describe the experience - did you discuss questions you might have had about something?**<br>● How was the communication? Interaction?	**Essential**<br>**Probes**
**Challenges & barriers**	**(4) Can anyone think of an instance when you felt you needed more information about something?**<br>● On a certain topic you were interested in or an issue that concerned you?	**Essential**<br>**Prompts**
**Sources and resources**	**(5) Other than talking to your health care provider or attending an antenatal class, what other sources of information are important?**<br>● Who do you turn to? Why?	**Essential**<br>**Prompts**
**Use of internet**	**(6) What about the internet? Describe your experience searching for information online.**<br>● What issues/topics did you search information for?<br>● How easy is/was it finding what you need? How did it make you feel?<br>● How do you assess how reliable the online information is?	**Essential**<br>**Prompts**<br>**Probe**
**User-provider communication and internet use**	**(7) Can you describe your experience talking to your provider (gynaecologist-obstetrician, midwife, paediatrician, other) about information you found on the internet?**<br>● Do they encourage or discourage the use of the internet? Can you think of some examples?<br>● Are they open and welcoming to such information? Do they discuss it with you?	**Essential**<br>**Prompts**<br>**Probes**
**Decision making, and satisfaction (general)**	**(8) What would you say helped you the most in terms of knowing your choices during the pregnancy, for birth or for caring for you baby?**<br>● Did you feel supported in terms of the information you had to know your choices or make decisions?	**Essential**<br>**Probes**
**Closing**	**(9) Is there anything else you might want to add or discuss with the group – anything, even if you don't think it's relevant, it might be helpful.**	**Essential**

© 2023 Koliandri I. et al.
